# *H. pylori* modulates DC functions via T4SS/TNFα/p38-dependent *SOCS3* expression

**DOI:** 10.1186/s12964-020-00655-1

**Published:** 2020-10-06

**Authors:** Muamera Sarajlic, Theresa Neuper, Julia Vetter, Susanne Schaller, Maria M. Klicznik, Iris K. Gratz, Silja Wessler, Gernot Posselt, Jutta Horejs-Hoeck

**Affiliations:** 1 Department of Biosciences, University of Salzburg, Hellbrunner Strasse 34, 5020 Salzburg, Austria; 2 Bioinformatics Research Group, University of Applied Sciences Upper Austria, Hagenberg im Muehlkreis, Austria

**Keywords:** Dendritic cell, SOCS, p38, *H. Pylori*, Type IV secretion system

## Abstract

**Background:**

*Helicobacter pylori* (*H. pylori*) is a gram-negative bacterium that chronically infects approximately 50% of the world’s human population. While in most cases the infection remains asymptomatic, 10% of infected individuals develop gastric pathologies and 1–3% progress to gastric cancer. Although *H. pylori* induces severe inflammatory responses, the host’s immune system fails to clear the pathogen and *H. pylori* can persist in the human stomach for decades. As suppressor of cytokine signaling (SOCS) proteins are important feedback regulators limiting inflammatory responses, we hypothesized that *H. pylori* could modulate the host’s immune responses by inducing SOCS expression.

**Methods:**

The phenotype of human monocyte-derived DCs (moDCs) infected with *H. pylori* was analyzed by flow cytometry and multiplex technology. SOCS expression levels were monitored by qPCR and signaling studies were conducted by means of Western blot. For functional studies, RNA interference-based silencing of *SOCS1–3* and co-cultures with CD4^+^ T cells were performed.

**Results:**

We show that *H. pylori* positive gastritis patients express significantly higher *SOCS3*, but not *SOCS1* and *SOCS2*, levels compared to *H. pylori* negative patients. Moreover, infection of human moDCs with *H. pylori* rapidly induces *SOCS3* expression, which requires the type IV secretion system (T4SS), release of TNFα, and signaling via the MAP kinase p38, but appears to be independent of TLR2, TLR4, MEK1/2 and STAT proteins. Silencing of *SOCS3* expression in moDCs prior to *H. pylori* infection resulted in increased release of both pro- and anti-inflammatory cytokines, upregulation of PD-L1, and decreased T-cell proliferation.

**Conclusions:**

This study shows that *H. pylori* induces SOCS3 via an autocrine loop involving the T4SS and TNFα and p38 signaling. Moreover, we demonstrate that high levels of SOCS3 in DCs dampen PD-L1 expression on DCs, which in turn drives T-cell proliferation.

Video Abstract

## Background

With more than 4 billion infected persons, *Helicobacter pylori* is one of the most prevalent human pathogens worldwide. *H. pylori* infection is characterized by persistent colonization of the gastric mucosa [1] associated with leukocyte infiltration and increased secretion of pro-inflammatory cytokines within the first 2 weeks of infection [2, 3]. Without antibiotic treatment, however, the host’s immune system fails to clear the bacterial burden and *H. pylori* infection lasts for the entire life of the host [4]. Therefore, infected individuals experience chronic infections which can give rise to severe gastritis, several ulcer entities and gastric cancer [5–7]. Accordingly, *H. pylori* was categorized as a class I (definite) carcinogen by the World Health Organization (WHO) in 1994 [8]. *H. pylori* harbors several virulence factors, including the vacuolating toxin VacA, the serine protease HtrA, and a pathogenicity island encoding a type IV secretion system (T4SS) which delivers bacterial factors directly into the host cell cytoplasm (cagPAI). These latter factors include the bacterial protein CagA, peptidoglycan, and ADP-glycero-β-D-manno-heptose (ADP heptose) and are thought to hijack host cell signaling networks [9, 10].

In stomach biopsies of *H. pylori*-infected individuals, increased numbers of tissue-infiltrating immune cells have been identified, including neutrophils [11], B and T cells [12], and dendritic cells (DCs) [13]. Moreover, it has been reported that *H. pylori* infection results in recruitment of myeloid DCs to the inflamed mucosa. In contrast, biopsies from uninfected individuals lack myeloid DCs [14]. Furthermore, DCs were shown to take up virulence products of *H. pylori* [15] and to play key roles in initiating adaptive immune responses toward *H. pylori* [16]. However, the situation in *H. pylori* infections is ambiguous. Despite effective evasion from Toll-like receptor-4- (TLR4) and TLR5-mediated pathogen recognition, significant DC activation is observed [17–19]. While the effects of *H. pylori* infection on epithelial cells have been extensively studied, the consequences for human DCs are less well characterized.

Stimulation of DCs with bacterial components results in DC activation and maturation, which involves a wide variety of signaling cascades and results in the secretion of pro-inflammatory mediators as well as presentation of processed antigen in the context of co-stimulatory molecules. Mature DCs thus provide important signals that determine the development of different pathogen-specific T-helper cell subgroups, which in turn are crucial for protective immunity. A strong inflammatory response ensures killing of pathogens; however, to avoid excessive inflammation, several mechanisms have evolved to tightly regulate these processes. A well-described group of immune regulators are the suppressor of cytokine signaling (SOCS) proteins [20]. The SOCS protein family consists of 8 members (SOCS1–7, CIS) which are upregulated upon stimulation of cytokine receptors and TLRs [21–23]. SOCS proteins inhibit immune signaling at different levels of signal transduction, including inhibition of receptor-associated kinases, competition with signal transducers and activators of transcription (STATs), and proteasomal degradation of signaling molecules. Hence, SOCS proteins play an important role in limiting excessive immune responses [21]. However, recent studies indicate that SOCS proteins not only regulate cytokine signaling, but also have a direct impact on the inflammatory potential of DCs [23, 24].

While several studies have described a pro-inflammatory role of DCs in *H. pylori* infection, other data suggest that *H. pylori*-infected DCs are more likely to have a regulatory phenotype [16]. Since SOCS proteins can shift the balance between the pro- and anti-inflammatory potential of DCs [23–28], and because the role of SOCS proteins during *H. pylori* infection has been underexplored, this study aimed to shed light on SOCS proteins as possible regulators of DC function in response to *H. pylori* infection. Here, we show that human DCs rapidly upregulate *SOCS3* expression after infection with wild-type *H. pylori* P12, whereas the induction of *SOCS1* and *SOCS2* expression is observed only at later time points. Immediate *H. pylori*-induced *SOCS3* expression appears to be independent of TLR, MEK and JAK signaling. Instead, we identified the T4SS and TNFα as being necessary for early activation of MAP kinase p38 and subsequent *SOCS3* expression. Silencing studies revealed that *SOCS3* expression limits *H. pylori*-induced secretion of pro- as well as anti-inflammatory cytokines by DCs while it specifically dampens PD-L1 expression but not co-stimulatory molecules. Accordingly, we demonstrate that SOCS expression in DCs is important for subsequent T-cell proliferation. Taken together, this study identifies SOCS3 as an important player in the induction of *H. pylori*-induced immune responses.

## Methods

### Generation of monocyte-derived dendritic cells (DCs)

Monocyte-derived dendritic cells (moDCs) were generated from monocytes isolated from fresh buffy coats of healthy, anonymous donors (Blood Bank Salzburg, Austria) as described previously [23]. Briefly, peripheral blood mononuclear cells (PBMCs) were isolated by density gradient centrifugation using Histopaque-1077 (Sigma). After erythrocyte lysis with ACK buffer (150 mM NH_4_Cl, 10 mM KHCO_3_, 0.1 mM EDTA, pH 7.4), monocytes were isolated by means of magnetic labeling using CD14 Microbeads Ultrapure (MACS Miltenyi) or by monocyte adherence (70 min at 37 °C, 5% CO_2_), and cultured in RPMI 1640 supplemented with 10% heat-inactivated fetal calf serum, 1% L-glutamine (PAA), 10 U/ml penicillin/streptomycin (Sigma), 50 μM β-mercaptoethanol (Gibco). Immature DCs were generated by supplementation of the medium with 50 ng/ml GM-CSF and IL-4, respectively, for 7 days. After 2 days of differentiation, fresh medium was added to the culture. After 7 days, DCs were harvested and seeded in 24-well plates for infection.

### Bacterial culture and infection experiments

*H. pylori* wild-type P12 (wt) and P12 ΔcagPAI were cultured at 37 °C on GC agar plates (10% horse serum) under microaerophilic conditions (CampyGen Atmosphere Generation Systems, Thermo Scientific). For P12 ΔcagPAI, selective GC agar plates supplemented with kanamycin (8 μg/ml) were used. For infection, *H. pylori* was harvested and added to the DCs at a multiplicity of infection (MOI) of 0.2–20 or MOI = 5, respectively, for the indicated time points. For TLR inhibition experiments, DCs were treated with α-TLR2 (PAb-hTLR2, InvivoGen) and α-TLR4 (W7C11, α-hTLR4-IgG, InvivoGen) (1 μg/ml each) 20 min prior to infection. As a control, DCs were stimulated with 1 ng/ml LPS (*E. coli*). For the MAPK inhibition experiments, cells were treated with specific inhibitors of MEK1/2 (U0126, InvivoGen) or p38 (SB203, InvivoGen) at a concentration of 10 μM 1 h prior to infection. JAK kinase inhibition was performed by addition of a specific inhibitor (CAS 457081–03-7, Calbiochem, 1 μg/ml) 20 min prior to infection. TNFα was neutralized by using a human neutralizing antibody (D1B4, Cell Signaling) at a concentration of 100 ng/ml.

### Flow cytometry and ELISA

DC phenotypes were determined by analysis of median fluorescence intensities of surface marker expression measured on a FACS Canto II flow cytometer (BD Biosciences). Cells were harvested and resuspended in FACS buffer (PBS, 1% BSA, 2 mM EDTA) before staining (30 min, 4 °C, dark) using the following antibodies: CD1a-BV421 (HI149), CD40-FITC (SC3) and CD86-PE (IT2.2) (eBioscience), CD80-APC-H7 (L307.4) and PD-L1-PE-Cy7 (MIH1) (BD Biosciences), HLA-DR-APC (LN3, Invitrogen), PD-L2-APC-Vio770 (Miltenyi) as well as LD (eBioscience Fixable Viability Dye eFluor 780 and eFluor 506). Analysis of the median fluorescence intensity of the mentioned markers within the living CD1a^+^ moDC population was performed with FlowJo Software. Cytokine ELISA in supernatants of infected cells was performed according to the manufacturer’s instructions: IL-1β (R&D) and IL-6, IL-8, IL-12, TNF-α (all Peprotech).

### Multiplex assay

Cytokine and chemokine secretion of infected DCs was analyzed using the Cytokine/Chemokine/Growth Factor 45-Plex Human ProcartaPlex™ from ThermoFisher. Briefly, beads were washed once (PBS, 0.05% Tween-20) and resuspended in assay buffer (PBS, 0.05% Tween-20, 1% heat-inactivated FCS) before 8.34 μl per well were added into a 96-well V-bottom plate. Subsequently, 15 μl of standard or samples were added and the plate was incubated on an orbital shaker at 4 °C overnight. The next day, samples were washed three times and resuspended in 15 μl of detection antibody solution and incubated for 30 min at room temperature. Samples were again washed three times before 20 μl of Streptavidin-PE solution (1:1 in assay buffer) were added to each well and incubated for 30 min at room temperature. Eventually, samples were washed three times and resuspended in drive fluid for analysis. Measurement was performed on a Luminex Magpix instrument and data were analyzed using Procarta Plex Analyst Software (ThermoFisher).

### RNA isolation and quantitative real-time PCR

Total RNA was isolated using TRI Reagent (Sigma) and reverse-transcribed with RevertAid H Minus M-MulV reverse transcriptase (Thermo Scientific) according to the manufacturer’s instructions. Expression levels were determined by quantitative real-time PCR on a Rotorgene 3000 (Corbett Research) using IQ SYBR Green Supermix. Levels of the large ribosomal protein P0 (RPLP0) served as a reference gene. Relative mRNA expression x was calculated using the equation x = 2^-ΔCt^, where Δct represents the difference between the threshold cycle (ct) of the gene of interest and the reference gene. PCR specificity was assessed by recording a melting curve for the PCR products. The following primer pairs were used for quantification: *SOCS1*: sense 5′-TTGGAGGGAGCGGATGGGTGTAG- 3′, antisense 5′-AGAGGTAGGAGGTGCGAGTTCAGGTC-3′; *SOCS2*: sense 5′-CCAAATCAACCAAAAAAAGTGACCATGAAGTCCTG-3′, antisense 5′- CGGGGATTGAGTTGCACCTGTATAGCATGATATTC-3′; *SOCS3*: sense 5′- ATACTATACCTTCCTGTACCTGGGTGGATGGAGCG-3′, antisense 5′- TGAGTATGTGGCTTTCCTATGCTGGGTCCCTCT-3′;

### Database analysis

For analysis of *SOCS* expression in gastritis patients, the public genomic dataset GSE5081 from NCBI Gene Expression Omnibus (NCBI-GEO) [29] was used. The data discussed in the present study were derived from Galamb et al. [30], have been deposited in NCBI-GEO, and are accessible through GEO Series accession number GSE5081. Analysis was performed using Python. Access to the GEO database was granted with the following GEOparse package (https://github.com/guma44/GEOparse). The GSE5081 dataset includes whole-genome oligonucleotide microarray analysis data of *H. pylori*-related (HP+) and idiopathic (HP-) gastritis patients. Gene expression data derive from total RNA which was extracted from frozen gastric biopsy specimens. The dataset includes 32 samples of 16 patients (8 HP+, 8 HP-), in which erosive and non-erosive (normal antrum) areas were used for biopsies. While *SOCS1* and *SOCS2* gene expression data show no statistical difference, *SOCS3* expression does show a statistically significant difference between HP+ and HP- samples (Affymetrix Probe Set IDs in Platform GPL570: 213337_s_at, 232539_at and 227697_at).

### Western blot

Cell pellets of infected samples were harvested and lysed in 80 μl 2x Laemmli sample buffer (BIO-RAD) supplemented with 5% β-mercaptoethanol (Sigma). Samples were separated on a 4–12% gradient gel (NuPAGE, Life Technologies) and blotted onto a nitrocellulose membrane (BIO-RAD). Membranes were blocked in TBS supplemented with 0.1% Tween and 5% nonfat dry milk for 1 h. The following antibodies were used according to the manufacturer’s instructions: p-STAT1-Tyr701 (Cat no. 5375S), STAT-1 (Cat no. 9172S), p-STAT3-Tyr705 (Cat no. 9145S), STAT3 (Cat no. 4904S), p-p38-Thr180/Tyr182 MAPK (Cat no. 9215S), p38 (Cat no. 9212S), and HRP-linked anti-rabbit secondary antibody (Cat no. 7074) (all Cell Signaling Technology). Detection was done using West Pico PLUS Chemiluminescent Substrate (Thermo Fisher) and BioMax films (Kodak). Blots were quantified using ImageJ (NIH) software [31].

### Gene silencing via siRNA-based transfection

DCs were transfected with small interfering RNAs (siRNAs) targeting *SOCS1* (Santa Cruz Biotechnology, Inc.), *SOCS2* (ThermoFisher), *SOCS3* (ThermoFisher) or Allstars negative control (Qiagen). For transfection, Lipofectamine RNAiMAX reagent (Life Technologies) was used according to the manufacturer’s instructions. In brief, 2 × 10^5^ DCs were seeded in 100 μl DC medium and transfected with 100 μl Opti-MEM (Life Technologies) containing 50 to 100 pmol siRNA and 1 μl transfection reagent. Subsequently, DCs were incubated for 6 h before 800 μl of fresh DC medium was added. After 48 h of incubation, DCs were used for infection experiments after silencing efficiency was assessed by qRT-PCR.

### Allogeneic moDC/T-cell co-culture

Control or *SOCS3*-silenced moDCs were harvested after 48 h and replated at a density of 10^5^ cells/400 μl in P/S free T-cell medium (IMDM + 5% FCS, 1x L-Glut). Thereafter, moDCs were either infected with *H. pylori* or left untreated for 24 h. The next day, 6 h pre addition of T cells, the cultures were supplemented with P/S (1x). Total CD4^+^ T cells were isolated from buffy coats of healthy donors using the Human CD4^+^ T cell isolation kit (Miltenyi Biotec) according to the manufacturer’s instructions, and isolated T cells were stained with e450 Proliferation Dye (eBioscience) for 10 min at room temperature. Thereafter, T cells were added to the moDC culture at a ratio of 1:10 (moDC:T cell). IL-2 was added at a concentration of 50 U/ml. After 6 days of co-culture, T cells were stimulated with PMA (50 ng/ml), Ionomycin (1 μg/ml) and Brefeldin A (10 μg/ml, Sigma-Aldrich B652) for 4 h. Thereafter, cells were transferred in a V-bottom plate and stained in 20 μl PBS containing the following antibodies for surface staining: CD4-PE-594 (RPT-4A, BioLegend) and LD (eBioscience Fixable Viability Dye eFluor 780) for 30 min (4 °C, dark). After washing of the samples (150 μl PBS), 100 μl Fix-Perm (FOXP3 staining kit, eBioscience) were added and the samples were incubated for 30 min (4 °C, dark). Meanwhile, the intracellular staining mix was prepared in PermBuffer using IFNγ-PE-Cy7 (B27). After two washing steps, IFNγ-PE-Cy7 (B27) diluted in PermBuffer was added to the samples for 20 min. Measurement was performed in PBS + 2 mM EDTA on a CytoFLEX S instrument (Beckman Coulter). Living, single, CD4^+^ T cells were used to analyze IFNγ levels.

### Statistics

Data are presented as bars indicating mean ± standard deviation (SD) or as dot plots. Statistical analyses were performed with GraphPad Prism 7 software. Multiple groups were analyzed by one-way ANOVA including a post-hoc test. *P* values < 0.05 were considered significant (*p < 0.05, **p < 0.01, ***p < 0.001, n.s. not significant).

## Results

### *H. pylori* induces cytokine secretion and expression of feedback inhibitors in a time-dependent way

*H. pylori* has been shown to penetrate the gastric lining and directly interact with cells of the immune system. Immature DCs are recruited to the inflamed mucosa where they encounter the bacteria and undergo maturation, which initiates an adaptive immune response toward the pathogen [32]. In general, the DC maturation process involves the release of inflammatory cytokines and elevated levels of co-stimulatory molecules, which are crucial for T-cell activation [33, 34]. Accordingly, we observed dose-dependent upregulation of activation markers as well as cytokine secretion upon *H. pylori* infection (Supplementary Figure 1). It is well established that bacterial stimuli also induce the expression of immunosuppressive molecules, including programmed death ligand 1 (PD-L1) and PD-L2, in order to prevent excessive damage to the tissue following an inflammatory response [35, 36]. Consistent with this, we observed significant and dose-dependent increases of both PD-L1 and PD-L2 levels upon *H. pylori* infection (Supplementary Figure 1A).

**Fig. 1:**
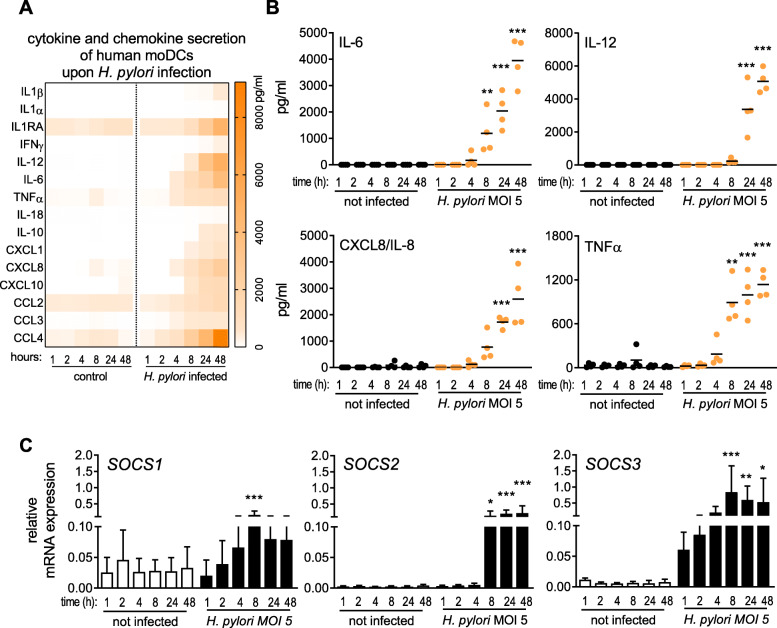
*H. pylori* infection elicits cytokine and chemokine secretion as well as SOCS expression in moDCs. DCs were re-plated in fresh medium, *H. pylori* strain P12 was harvested in PBS and added to the cells at MOI = 5. **a** Heatmap depicting cytokine and chemokine secretion by DCs infected for the indicated times, ranging from 1 to 48 h, analyzed by Multiplex Assay. **b** Detailed presentation of secretion of IL-6, IL-8, IL-12 and TNFα by infected moDCs in a time-dependent manner. Each dot represents an individual donor; the horizontal line in each column indicates the mean value of one experiment comprising 4 donors. (*N* = 4) **c** *SOCS1*, *SOCS2*, *SOCS3* mRNA expression was monitored by qRT-PCR. Bars represent mean + SD of three experiments comprising six individual donors. (*N* = 6) For statistical analysis, one-way ANOVA with a Sidak post-hoc test was performed. *p < 0.05, **p < 0.01, ***p < 0.001

**Fig. 2:**
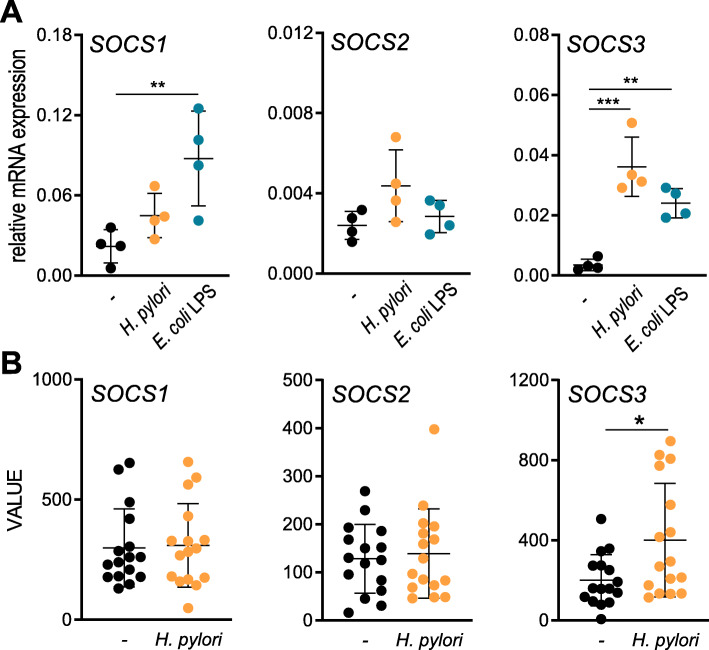
*H. pylori* selectively augments *SOCS3* expression. **a** DCs were infected with *H. pylori* strain P12 at MOI = 5 or stimulated with *E. coli*-derived LPS (1 ng/ml) for 4 h. SOCS mRNA expression was measured by qRT-PCR. Dots represent individual donors; the horizontal line in each column indicates the mean value ± SD of one experiment comprising four donors. (*N* = 4) For statistical analysis, one-way ANOVA with Tukey’s post-hoc test was performed. **p < 0.01, ***p < 0.001 **b** SOCS expression in *H. pylori*-negative and *H. pylori*-positive gastritis patients was determined using a publicly available genomic dataset (GSE5081), which was analyzed using Python. Dots represent individual donors; the horizontal line in each column indicates the mean value ± SD. (*N* = 16) For statistical analysis, a two-tailed, unpaired t test was performed. *p < 0.05

To examine the inflammatory response of DCs to *H. pylori* infection in more detail, we monitored cytokine and chemokine secretion upon infection over a defined time period (Fig. 1a). We observed the release of a wide variety of mediators, with IL-6 and TNFα being significantly increased at 8 h post-infection with *H. pylori*, whereas most other cytokines were detected only after 24 h (Fig. 1a/b). In light of these strong increases in cytokine levels, we additionally investigated the mRNA expression of suppressor of cytokine signaling (SOCS) proteins SOCS1, − 2 and − 3 over a period of 48 h post *H. pylori* infection (Fig. 1c). Our data reveal that *SOCS3* mRNA was already induced within 1 h of infection, whereas *SOCS1* and *SOCS2* expression was delayed and detectable only after 8 h. Neither *H. pylori* infection nor TLR4 stimulation with *E. coli* LPS was able to induce *SOCS2* expression within 4 h of treatment (Fig. 2a), which is in line with previous studies demonstrating the delayed expression profile of *SOCS2* [23]. Interestingly, LPS significantly induced the expression of both *SOCS1* and *SOCS3* at this early time point, whereas *H. pylori* selectively increased only *SOCS3* expression (Fig. 2a). Additionally, we analyzed SOCS expression in a data set of *H. pylori*-positive and negative human gastric biopsies and found that *SOCS3* was significantly increased in *H. pylori*-positive samples compared to *H. pylori*-negative ones, while *SOCS1* and *SOCS2* were not differentially expressed (Fig. 2b). The exclusive and rapid induction of *SOCS3* suggests that *SOCS3* might play a specific role in regulating DC activation in response to *H. pylori* infection.

### *H. pylori* triggers early *SOCS3* expression via p38 signaling but independent of TLR, MEK1/2 and JAK

**Fig. 3:**
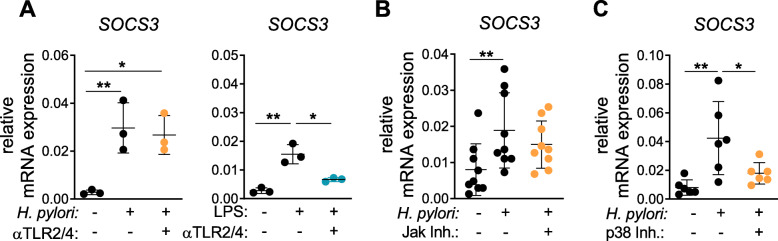
*H. pylori*-induced *SOCS3* expression is mediated by p38 and independent of TLR2/4 and JAK kinase. After 1 h of infection with *H. pylori* at MOI = 5 or stimulation with LPS, DCs were harvested and *SOCS3* mRNA expression was analyzed by qRT-PCR. **a** 20 min prior to infection with *H. pylori* or stimulation with LPS (1 ng/mL), blocking antibodies for TLR2 (1 μg/mL) and TLR4 (1 μg/mL) were added to the cells (αTLR2/4). *H. pylori* strain P12 was added to the cells at MOI = 5 (orange). LPS was added at 1 ng/mL (blue). One experiment comprising 3 individual donors is shown. (*N* = 3**) b, c** Specific inhibitors of JAK kinase (1 μg/ml) or p38 (SB203) (10 μM) were added to the respective samples. Dots represent individual donors; mean ± SD is shown. **b** Four experiments comprising 9 donors (*N* = 9) and **c** three experiments comprising 6 donors (*N* = 6) are shown. For statistical analysis, one-way ANOVA with Tukey’s post-hoc test was performed. *p < 0.05, **p < 0.01

To determine how *H. pylori* induces *SOCS3* expression within 1 h of infection, we first investigated whether activation of TLRs might contribute to this gene activation. In particular, TLR2 and TLR4 have been reported to be involved in sensing *H. pylori* products, which in turn leads to DC activation [19]. Therefore, we treated DCs with a combination of α-TLR2 and α-TLR4 blocking antibodies prior to infection and monitored subsequent expression of SOCS proteins. To control for successful TLR blocking, DCs were treated with *E. coli* LPS, a well-described ligand of TLR2 and TLR4 [37, 38]. Accordingly, we confirmed that addition of TLR2/4 blocking antibodies potently blocked *E. coli* LPS-induced mRNA expression of *SOCS3* by DCs (Fig. 3a, right panel). In contrast, *H. pylori*-induced *SOCS3* expression was not affected by the addition of the TLR blocking antibodies (Fig. 3a, left panel). These data demonstrate that, within the first hour of infection, *H. pylori*-induced *SOCS3* expression was independent of TLR2/4 signaling. Because JAK activation and subsequent STAT signaling are well-described mechanisms driving SOCS expression, we next determined whether this pathway may contribute to early *SOCS3* regulation [39]. Similar to our previous inhibition experiments, JAK inhibition did not have an impact on *H. pylori*-induced *SOCS3* expression (Fig. 3b). Therefore, we focused on mitogen-activated protein (MAP) kinases, which have been shown to be rapidly activated by *H. pylori* in epithelial cells [40] and to play a role in regulation of *SOCS3* mRNA levels [41]. To test whether these kinases are potential inducers of *SOCS3* in DCs, we blocked p38 and MEK1/2 prior to infection and monitored *SOCS3* expression 1 h post infection. While inhibition of MEK1/2 had no effect on *SOCS3* induction (Supplementary Figure 2), blocking of p38 decreased the capacity of *H. pylori* to induce *SOCS3* (Fig. 3c). These data suggest that MAPK p38 is crucially involved in *H. pylori*-induced *SOCS3* expression within the first hour of infection.

### *H. pylori* wild type but not the ΔCagPAI mutant induces *SOCS3* expression via p38

**Fig. 4:**
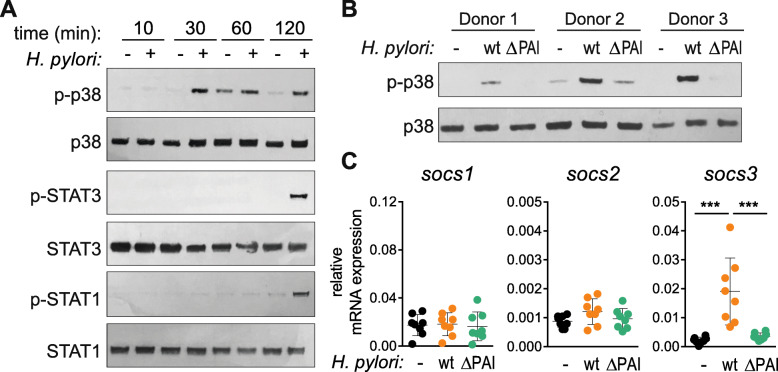
*H. pylori* triggers early phosphorylation of p38 which correlates with *SOCS3* expression. Day-7 DCs were re-plated in fresh medium, *H. pylori* strain P12 was harvested in PBS and added to the cells at MOI = 5. **a** Protein levels and phosphorylation of p38, STAT1 and STAT3 were analyzed at the indicated time points by western blotting. One representative experiment out of two is shown. **b** DCs were infected with *H. pylori* strain P12 wt or ΔCagPAI, and p-p38 levels were detected after 30 min. Three individual donors are shown. **c** DCs were infected with *H. pylori* strain P12 wt or ΔCagPAI, and *SOCS3* mRNA expression was analyzed after 1 h. Dots represent individual donors, mean ± SD of eight individual donors is shown. For statistical analysis, one-way ANOVA with Tukey’s post-hoc test was performed. ***p < 0.001

To confirm the role of MAPK p38 in *H. pylori*-induced *SOCS3* expression, we infected DCs and monitored the phosphorylation of p38 (p-p38) during the first 2 h of *H. pylori* infection. We detected phosphorylated p38 as early as 30 min post-infection, whereas phosphorylation of the canonical *SOCS3*-inducer STAT3 [42] as well as STAT1 was observed only after 2 h (Fig. 4a). This further supports our hypothesis that the p38 MAPK pathway is involved in *H. pylori*-induced *SOCS3* expression, whereas STAT3 plays a minor role in this context. Because we already excluded TLR2 and TLR4 as triggers of early *SOCS3* expression (Fig. 3a), we next addressed the question of whether the CagPAI-encoded type IV secretion system (T4SS) is necessary for p38 phosphorylation and thus for early induction of *SOCS3*. We infected DCs of three independent donors with either the *H. pylori* wild-type (wt) strain P12 or a P12 mutant lacking CagPAI (ΔCagPAI) and analyzed p38 phosphorylation. While the *H. pylori* wt strain strongly induced phosphorylation of p38, the T4SS-deficient strain ΔCagPAI showed severely impaired p38 activation (Fig. 4b). We additionally analyzed *SOCS3* expression in cells infected with *H. pylori* wt or the ΔCagPAI mutant and observed significantly lower *SOCS3* mRNA levels upon infection with the mutant compared with the wt strain (Fig. 4c). These results indicate that both the T4SS and p38 are necessary for *H. pylori*-induced early *SOCS3* expression.

### Early *SOCS3* expression is a consequence of T4SS-dependent release of TNFα

**Fig. 5:**
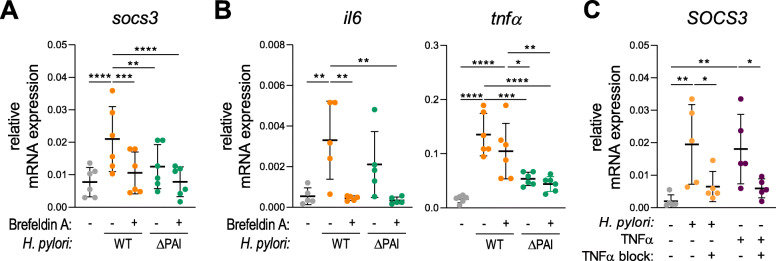
*SOCS3* expression is a secondary response to *H. pylori*-induced TNFα. After 1 h of infection with *H. pylori* at MOI = 5, DCs were harvested and mRNA expression was analyzed by qRT-PCR. **a, b** 1 h prior to infection with *H. pylori* strain P12 WT (orange) or ΔCagPAI (green), Brefeldin A (1 μg/ml) was added to the cells. *H. pylori* was added to the cells at MOI = 5 and *SOCS3*, *IL6* and *TNFα* mRNA expression was evaluated after 1 h. Two experiments comprising 6 individual donors (N = 6) are shown. Dots represent individual donors; mean ± SD is shown. **c** Cells were treated with a specific TNFα neutralizing antibody (100 ng/ml) before infection with *H. pylori* strain P12 WT or stimulation with TNFα (10 ng/ml). After 1 h, *SOCS3* mRNA expression was assessed. One experiment comprising 5 individual donors (*N* = 5) is shown. Dots represent individual donors; mean ± SD is shown. For statistical analysis, one-way ANOVA with Tukey’s post-hoc test was performed. *p < 0.05, **p < 0.01

As a next step we investigated whether *H. pylori* infection per se or mediators released upon *H. pylori* infection drive *SOCS3* expression. Interestingly, we observed that *H. pylori* wt-induced *SOCS3* expression was significantly inhibited when cytokine release was blocked using Brefeldin A (Fig. 5a). Treatment with Brefeldin A results in *SOCS3* expression comparable to that observed upon infection with the ΔCagPAI mutant. This suggests that infection with the ΔCagPAI mutant does not induce the secretion of the soluble mediator crucial for *SOCS3* expression (Fig. 5a). As IL-6 and TNFα are both known to enhance *SOCS3* expression, we monitored gene expression of these cytokines in the same experimental setting. Intriguingly, the ΔCagPAI mutant was able to induce expression of IL-6 but failed to induce expression of TNFα after 1 h of infection, whereas Brefeldin A treatment resulted in decreased IL-6 but stable TNFα mRNA levels upon infection with the wt strain (Fig. 5b). These results suggest a potential role for TNFα in mediating *H. pylori*-induced *SOCS3* mRNA expression. To confirm this hypothesis, we induced moDCs with TNFα to analyze its capacity to induce *SOCS3* mRNA expression and, additionally, we blocked TNFα in DCs using a neutralizing antibody prior to infection. We found that TNFα stimulation results in *SOCS3* mRNA expression, which could be blocked upon neutralization of TNFα (Fig. 5c). Interestingly, blocking of TNFα also suppressed *H. pylori* wt-induced *SOCS3* expression (Fig. 5c). Taken together, this set of data suggests that only *H. pylori* wt, but not the ΔCagPAI mutant, is able to induce TNFα expression within 1 h of infection, which in turn activates the expression of *SOCS3* mRNA.

### Silencing of *SOCS3* in DCs results in enhanced PD-L1 expression and dampened CD4^+^ T-cell proliferation

**Fig. 6:**
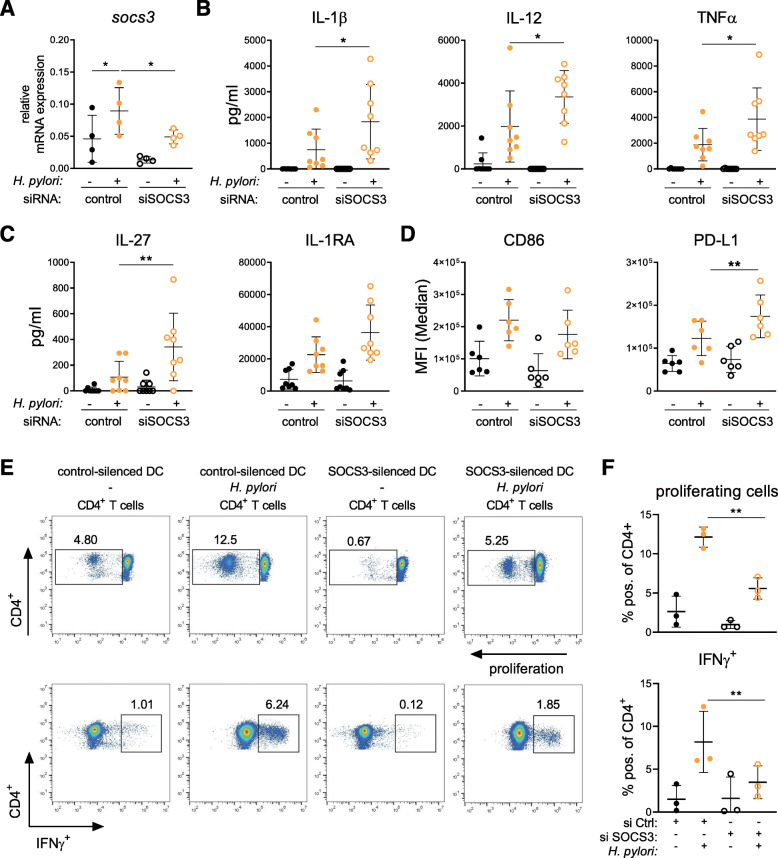
*SOCS3* silencing shapes *H. pylori*-induced DC activation and promotes proliferation of T cells. Immature day-7 DCs were re-plated and transfected with siRNA targeting *SOCS3* or non-targeting control oligo (both 100 pmol). 48 h post-transfection, *H. pylori* strain P12 was harvested in PBS and added to the cells at MOI = 5. **a** Silencing efficiency was analyzed by qRT-PCR after 2 h of infection. One experiment comprising four individual donors is shown. (*N* = 4) **b/c** Cytokine secretion of three experiments comprising eight donors (*N* = 8) was analyzed 24 h post-infection. **d** Surface marker expression of two experiments comprising six donors (*N* = 6) was analyzed 24 h after bacterial infection. **e/f** Control (si Ctrl) or *SOCS3*-silenced (si *SOCS3*) DCs were infected with *H. pylori* (MOI = 5) for 24 h before P/S was added for 6 h. Total CD4+ T cells were magnetically isolated from PBMCs and cultivated with DCs at a ratio of 1:10 (DC:T cell) for 6 days. On day 6, cells were restimulated with PMA/Ionomycin for 4 h and analyzed by means of flow cytometry. Dots represent individual donors (*N* = 3); mean ± SD is shown. For statistical analysis, one-way ANOVA with Tukey’s post-hoc test was performed. *p < 0.05, **p < 0.01

Having shown that *H. pylori* induces the expression of *SOCS3* within the first hour post infection, we analyzed whether the presence of *SOCS3* affects *H. pylori*-induced activation of human DCs. To investigate the role of *SOCS3* during *H. pylori* infection, we performed RNA interference-based silencing of *SOCS3* expression prior to infection. Transfection with siRNA targeting *SOCS3* effectively reduced *SOCS3* expression in DCs infected with *H. pylori*, compared to DCs treated with a non-targeting control siRNA (Fig. 6a). We then measured *H. pylori*-induced secretion of soluble mediators by DCs. The secretion of several pro-inflammatory and anti-inflammatory cytokines, including IL-1β, IL-12 and TNFα, IL-27 and IL-1RA, was augmented upon *H. pylori* infection of DCs transfected with siRNA targeting *SOCS3* (Fig. 6b/c). Interestingly, *SOCS3* silencing also resulted in a significant increase of PD-L1, whereas CD40, CD80, CD86 and PD-L2 remained unchanged, compared to control cells (Fig. 6d and Supplementary Figure 3). In contrast, silencing of *SOCS1* and *SOCS2*, respectively, did not significantly alter the DC phenotype upon infection (Supplementary Figure 4). These data indicate that *H. pylori*-induced *SOCS3* regulates inflammatory cytokine release, but it also dampens the secretion of anti-inflammatory mediators and the induction of the co-inhibitory molecule PD-L1, which could result in persistent inflammation. Therefore, the question arose whether *SOCS3* expression in DCs also boosts *H. pylori*-induced T-cell responses. To address this question, we performed allogeneic co-cultures with *SOCS3*-silenced moDCs and CD4^+^ T cells and analyzed proliferation as well as IFNγ expression. We observed significantly decreased T-cell proliferation and IFNγ expression by CD4^+^ T cells upon co-culture with *SOCS3*-silenced *H. pylori-*infected cells compared to control silenced cells. Therefore, these data indicate that *H. pylori* induced-*SOCS3* expression in DCs limits PD-L1 levels, which in turn results in increased T-cell proliferation.

## Discussion

Numerous studies have shown that *H. pylori* and soluble factors associated with this pathogen are potent activators of innate immune cells, as characterized by the release of pro-inflammatory mediators and expression of co-stimulatory molecules [43–45]. However, the immune response toward *H. pylori* fails to effectively clear the pathogen, leading to lifelong infection within the human stomach. Manifold evolved strategies to escape and manipulate the host’s innate and adaptive immunity make *H. pylori* a very successful pathogen. These mechanisms range from circumventing phagocytic activity and recognition by innate immune cells [17, 46] to inducing adaptive immune responses with a regulatory profile [47–49].

Although it is well established that immune cells drive the expression of negative feedback regulators (e.g. SOCS proteins) to limit excessive immune responses upon bacterial encounters [50, 51], evidence for a role of SOCS proteins during *H. pylori* infection remains scarce. As we are interested in the early events in DC activation critical for priming the signaling network of these cells, we investigated the transcriptional activation of negative feedback inhibitors at early time points after *H. pylori* infection. Our data indicate a specific and strong upregulation of *SOCS3* after 1 h of infection, while *SOCS1* and *SOCS2* displayed delayed kinetics. To identify the pathway involved in *SOCS3* transcription, we analyzed TLR signaling as well as the activation of MAPKs and STAT proteins, as both p38 and STAT3 have been linked to transcriptional regulation of *SOCS3* [42, 52, 53]. We demonstrated that early induction of *SOCS3* by *H. pylori* is dependent on an intact T4SS as well as MAPK p38, but independent of TLR2, TLR4, MEK1/2 and the JAK/STAT pathway. This raises the question whether p38 phosphorylation and *SOCS3* expression are stimulated by a specific cytokine milieu created immediately upon infection with *H. pylori* wt. Accordingly, we could demonstrate that *H. pylori* wt, but not the ΔCagPAI mutant, directly activates transcription of TNFα, which in turn results in upregulation of *SOCS3*. These data are in line with a study describing that p38 activation via TNFα enhances *SOCS3* mRNA stabilization and thus its expression in murine fibroblasts [41]. However, it remains unclear whether TNFα expression, and thus p38-mediated *SOCS3* expression, is stimulated by structural components of the T4SS or by injected factors*.* In this regard, quantitative phospho-proteomics data suggest that phosphorylation of p38 is independent of cagA translocation [54]. Accordingly, newer studies show that the LPS heptose core molecule ADP heptose is also injected via the T4SS into infected cells and triggers TIFA-dependent p38 signaling [55, 56].

As mentioned above, *H. pylori* specifically increases *SOCS3* at early time points. This is in contrast to stimulation of DCs with conventional *E. coli* LPS, where we observed rapid upregulation of both *SOCS1* and *SOCS3*. The synchronized regulation of *SOCS1* and *SOCS3* expression in DCs and macrophages has been reported by numerous studies employing purified TLR agonists, like LPS or CpG [57, 58], but also in response to bacterial infection with pathogens, including *Salmonella enterica, Chlamydia pneumoniae* and *Mycobacterium tuberculosis* [59–61]. In this respect, *SOCS1* expression in macrophages has been implicated in hampering of bacterial clearance during *Mycobacterium tuberculosis* and *Chlamydia pneumoniae* infection while also protecting the host from excessive pathogen-induced inflammation [61]. However, the role of *SOCS3* in innate inflammation is less well understood and our observation that *H. pylori* specifically induces *SOCS3* expression raises the question of the consequences of selective *SOCS3* expression. Murine bone-marrow-derived DCs overexpressing *SOCS3* were shown to express low levels of MHC II and CD86, and low levels of IL-12 and IL-23, upon stimulation with LPS [28]. Consistent with this, we showed that human DCs lacking *SOCS3* secrete higher amounts of IL-12, IL-23 and IL-1β, while we also detected increased expression of the anti-inflammatory cytokine IL-1RA. We also observed increased *H. pylori*-induced secretion of TNFα and IL-27 as well as enhanced levels of PD-L1 upon *SOCS3* silencing. The upregulation of PD-L1 might be a result of pronounced TNFα and/or IL-27 secretion, as recent studies described that both cytokines support PD-L1 expression [62, 63]. Whereas TNFα was shown to enhance PD-L1, but not PD-L2, in a NF-κB-dependent manner [62], IL-27 stimulation was found to be related to PD-L1 upregulation in various immune cells, including human monocytes and DCs [63]. Additionally, *SOCS3* overexpression has been reported to result in decreased levels of IL-27-induced PD-L1 expression [64], which suggests that *SOCS3* plays a role in limiting PD-L1 expression. To test whether DC-specific *SOCS3* deficiency also influences consequential T-cell responses, we performed co-culture experiments where we observed decreased T-cell proliferation as well as decreased IFNγ secretion by CD4^+^ T cells upon *SOCS3* silencing. This observation is in line with a study from Gao et al., who demonstrated that in a model of *Mycobacterium tuberculosis* infection, antigen-presenting cells lacking *SOCS3* were poor inducers of mycobacterial-specific T-cell responses [65].

## Conclusions

In conclusion, we demonstrated that *H. pylori* regulates *SOCS3* during early infection and that this process requires an intact T4SS system and TNFα-mediated activation of p38. Our results further suggest that hijacking of DC signaling cascades and consequential upregulation of *SOCS3* modulates T-cell responses upon *H. pylori* infection.

## Supplementary information

**Additional file 1 Figure S1**. *H. pylori* induces maturation of human DCs. Monocytes were isolated from human PBMCs and differentiated into moDCs in the presence of IL-4 and GMCSF (50 ng/mL each). Immature DCs were re-plated in fresh medium on day 7, *H. pylori* strain P12 was harvested in PBS and added to the cells at increasing MOIs of 0.2, 2 and 20. Surface marker expression **(A)** and cytokine secretion **(B)** were analyzed 48 h post-infection. Data represent mean + SD of one experiment comprising three individual donors (*N* = 3). For statistical analysis, one-way ANOVA with Dunnett’s post-hoc test was performed. *p < 0.05, **p < 0.01, ***p < 0.001. **Figure S2.** *H. pylori*-induced *SOCS3* expression is independent of MEK signaling. Cells were treated with a specific inhibitor of MEK kinase 20 min prior to infection with H. pylori wild-type strain P12 (MOI = 5). After 1 h of infection, DCs were harvested and *SOCS3* mRNA expression was analyzed by qRT-PCR. Two experiments comprising 4 donors (*N* = 4) are shown. For statistical analysis, one-way ANOVA with Tukey’s post-hoc test was performed. *p < 0.05, **p < 0.01. **Figure S3.** SOCS3 silencing does not affect chemokine secretion. Immature day-7 DCs were re-plated and transfected with siRNA targeting *SOCS3* or non-targeting control oligo (both 100 pmol). 48 h post-transfection, *H. pylori* strain P12 was harvested in PBS and added to the cells at MOI = 5. Surface marker expression **(A)** and chemokine secretion **(B)** were analyzed 24 h after bacterial infection. Three experiments comprising eight donors are shown. Dots represent individual donors; mean ± SD is shown. **Figure S4.** *SOCS1* and *SOCS2* silencing do not significantly alter DC activation. Immature day-7 DCs were re-plated and transfected with siRNA targeting *SOCS1* (50–100 pmol), *SOCS2* (100 pmol) or non-targeting control oligo (100 pmol). 48 h post-transfection, *H. pylori* strain P12 was harvested in PBS and added to the cells at MOI = 5. **(A,C)** Silencing efficiency was analyzed by qRT-PCR after 8 h of infection. **(B,D)** Surface marker expression was analyzed 24 h after bacterial infection. Three experiments comprising four donors (N = 4) **(A,B)** and three experiments comprising five donors (*N* = 5) **(C,D)** are shown. Dots represent individual donors; mean ± SD is shown. For statistical analysis, one-way ANOVA with Tukey’s post-hoc test was performed. *p < 0.05, **p < 0.01.

## Data Availability

The datasets used and/or analyzed in the present study are available from the corresponding author on reasonable request.

## Abbreviations

DC: Dendritic cell
MAPK: Mitogen-activated protein kinase
moDC: Monocyte-derived dendritic cell
MOI: Multiplicity of infection
PBMC: Peripheral blood mononuclear cell
PD-L1/2: Programmed death ligand 1/2
SOCS: Suppressor of cytokine signaling
STAT: Signal transducer and activator of transcription
TLR: Toll-like receptor
T4SS: Type IV secretion system
