# Supplementary material for: H. pylori modulates DC functions via T4SS/TNFα/p38-dependent SOCS3 expression
Source: Cell Commun Signal. 2020 Oct 6;18:160. doi: 10.1186/s12964-020-00655-1 (PMC7541176; doi:10.1186/s12964-020-00655-1)
Supplement: Supplementary file 2 — Additional file 1 Figure S1. H. pylori induces maturation of human DCs. Monocytes were isolated from human PBMCs and differentiated into moDCs in the presence of IL-4 and GMCSF (50 ng/mL each). Immature DCs were re-plated in fresh medium on day 7, H. pylori strain P12 was harvested in PBS and added to the cells at increasing MOIs of 0.2, 2 and 20. Surface marker expression (A) and cytokine secretion (B) were analyzed 48 h post-infection. Data represent mean + SD of one experiment comprising three individual donors (N = 3). For statistical analysis, one-way ANOVA with Dunnett’s post-hoc test was performed. *p < 0.05, **p < 0.01, ***p < 0.001. Figure S2. H. pylori-induced SOCS3 expression is independent of MEK signaling. Cells were treated with a specific inhibitor of MEK kinase 20 min prior to infection with H. pylori wild-type strain P12 (MOI = 5). After 1 h of infection, DCs were harvested and SOCS3 mRNA expression was analyzed by qRT-PCR. Two experiments comprising 4 donors (N = 4) are shown. For statistical analysis, one-way ANOVA with Tukey’s post-hoc test was performed. *p < 0.05, **p < 0.01. Figure S3. SOCS3 silencing does not affect chemokine secretion. Immature day-7 DCs were re-plated and transfected with siRNA targeting SOCS3 or non-targeting control oligo (both 100 pmol). 48 h post-transfection, H. pylori strain P12 was harvested in PBS and added to the cells at MOI = 5. Surface marker expression (A) and chemokine secretion (B) were analyzed 24 h after bacterial infection. Three experiments comprising eight donors are shown. Dots represent individual donors; mean ± SD is shown. Figure S4. SOCS1 and SOCS2 silencing do not significantly alter DC activation. Immature day-7 DCs were re-plated and transfected with siRNA targeting SOCS1 (50–100 pmol), SOCS2 (100 pmol) or non-targeting control oligo (100 pmol). 48 h post-transfection, H. pylori strain P12 was harvested in PBS and added to the cells at MOI = 5. (A,C) Silencing efficiency was analyzed by qRT-PCR a [file 12964_2020_655_MOESM2_ESM.pdf]

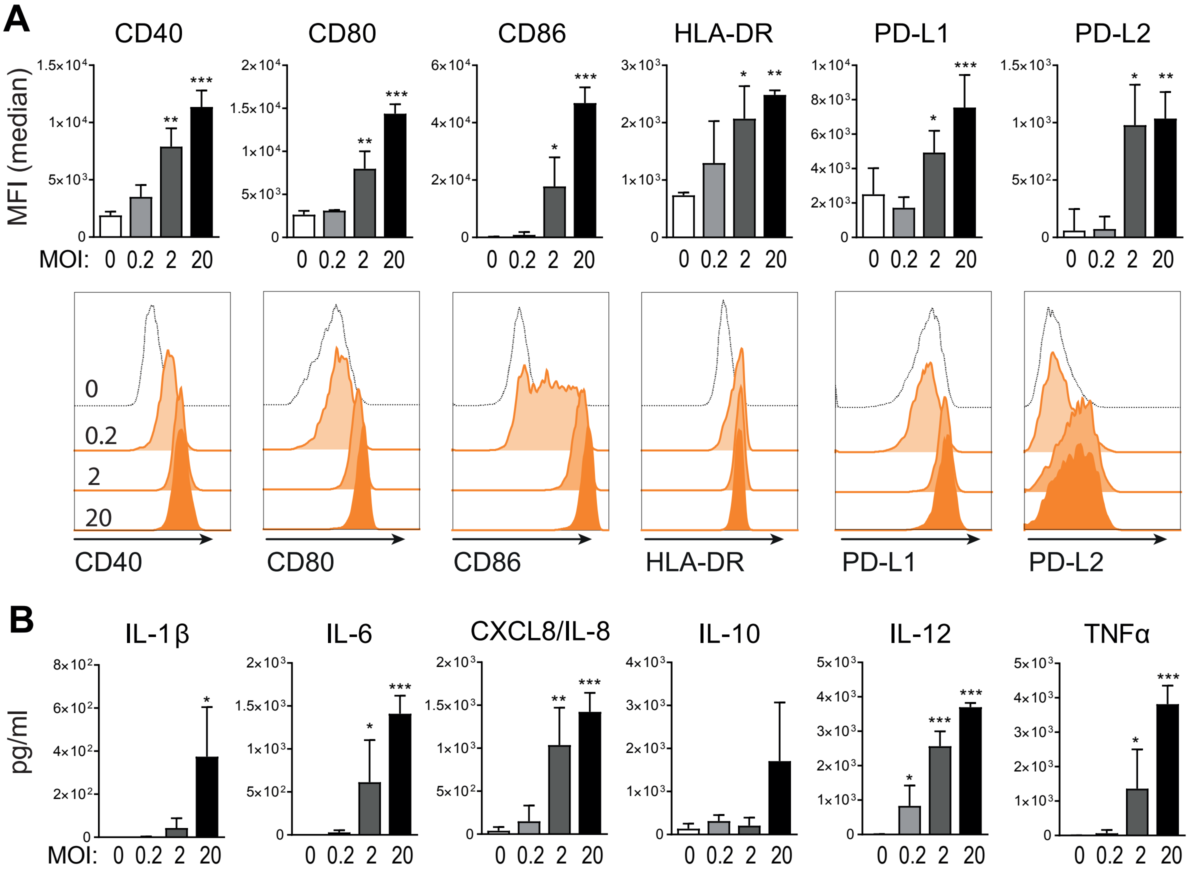

Supplementary Figure 1. *H. pylori* induces maturation of human DCs. Monocytes were isolated from human PBMCs and differentiated into moDCs in the presence of IL-4 and GM-CSF (50 ng/mL each). Immature DCs were re-plated in fresh medium on day 7, *H. pylori* strain P12 was harvested in PBS and added to the cells at increasing MOIs of 0.2, 2 and 20. Surface marker expression **(A)** and cytokine secretion **(B)** were analyzed 48 hours post-infection. Data represent mean+SD of one experiment comprising three individual donors (N=3). For statistical analysis, one-way ANOVA with Dunnett's post-hoc test was performed. *$p<0.05$, **$p<0.01$, ***$p<0.001$

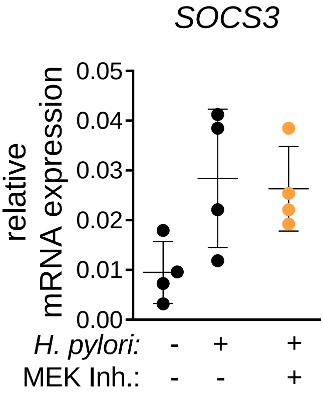

Supplementary Figure 2. *H. pylori*-induced *SOCS3* expression is independent of MEK signaling. Cells were treated with a specific inhibitor of MEK kinase 20 minutes prior to infection with H. pylori wild-type strain P12 (MOI=5). After 1 hour of infection, DCs were harvested and *SOCS3* mRNA expression was analyzed by qRT-PCR. Two experiments comprising 4 donors (N=4) are shown. For statistical analysis, one-way ANOVA with Tukey's post-hoc test was performed. *p<0.05, **p<0.01

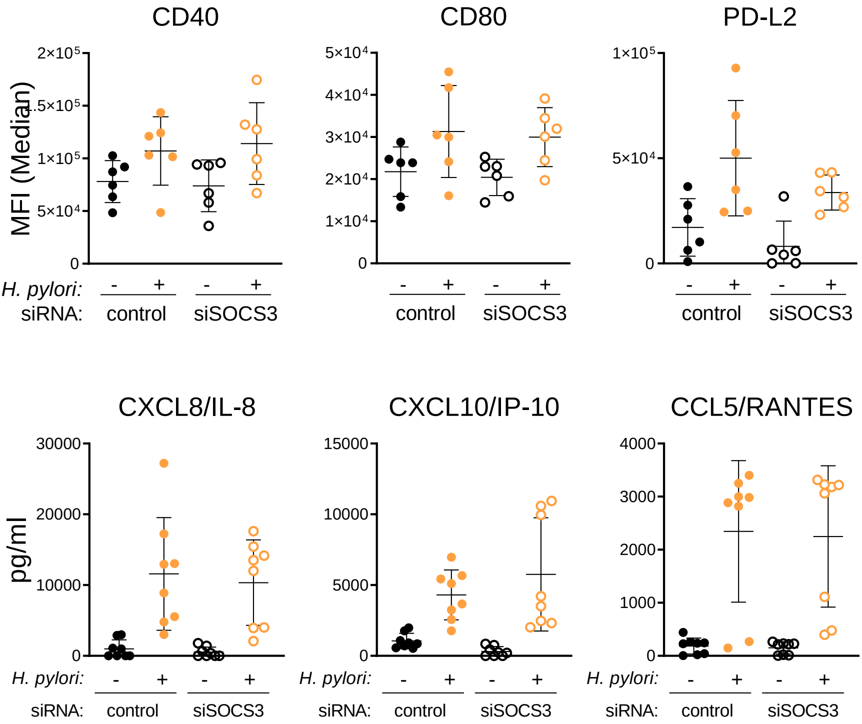

Supplementary Figure 3. SOCS3 silencing does not affect chemokine secretion. Immature day-7 DCs were re-plated and transfected with siRNA targeting *SOCS3* or non-targeting control oligo (both 100 pmol). 48 hours post-transfection, *H. pylori* strain P12 was harvested in PBS and added to the cells at MOI = 5. Surface marker expression **(A)** and chemokine secretion **(B)** were analyzed 24 hours after bacterial infection. Three experiments comprising eight donors are shown. Dots represent individual donors; mean ± SD is shown.

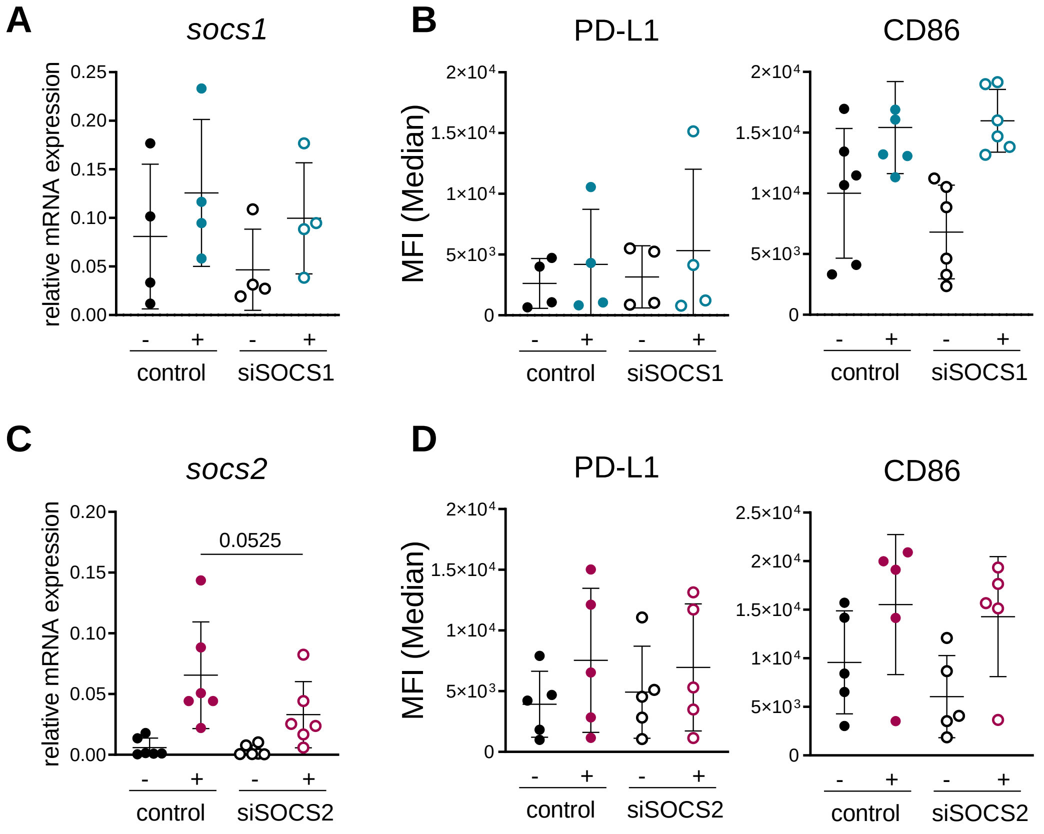

Supplementary Figure 4. *SOCS1* and *SOCS2* silencing do not significantly alter DC activation. Immature day-7 DCs were re-plated and transfected with siRNA targeting *SOCS1* (50-100 pmol), *SOCS2* (100 pmol) or non-targeting control oligo (100 pmol). 48 hours post-transfection, *H. pylori* strain P12 was harvested in PBS and added to the cells at MOI = 5. **(A,C)** Silencing efficiency was analyzed by qRT-PCR after 8 hours of infection. **(B,D)** Surface marker expression was analyzed 24 hours after bacterial infection. Three experiments comprising four donors (N=4) **(A,B)** and three experiments comprising five donors (N=5) **(C,D)** are shown. Dots represent individual donors; mean ± SD is shown. For statistical analysis, one-way ANOVA with Tukey's post-hoc test was performed. *$p<0.05$, **$p<0.01$
